# Oversight of bank risk-taking by audit committees and Sharia committees: conventional vs Islamic banks

**DOI:** 10.1016/j.heliyon.2021.e07798

**Published:** 2021-08-14

**Authors:** Quang Khai Nguyen

**Affiliations:** Shool of Banking, University of Economics, Ho Chi Minh City, Viet Nam

**Keywords:** Sharia committee, Audit committee, Bank risk-taking, Conventional bank, Islamic bank

## Abstract

By utilizing the Fixed effect and GMM estimators for a sample of 57 Islamic banks and 102 conventional banks from 10 countries for the period 2002–2018, we examine the effect of the audit committees' and Sharia committees' effectiveness on the bank risk-taking behavior and its transmission mechanisms. The results reveal that an audit committee's independence, number of meetings, and financial expertise negatively affect conventional banks' risk-taking, suggesting that the high effectiveness of their audit committees may constrain banks' risk-taking activities. However, no such relationship is evident or observed case of Islamic banks. Instead, with a different transmission mechanism, the proportion of female members and the financial expertise in the Sharia committees negatively affect risk-taking, but the Sharia committee size positively affects risk-taking in Sharia banks. These results indicate that a Sharia committee's high effectiveness can constrain risk-taking behaviors in Islamic banks.

## 1 Introduction

After the 2008 financial crisis, the role of banks in corporate governance, as overseeing and restricting risk-taking becomes a concern (Pathan, 2009; Sun and Liu, 2014; Nguyen, 2020). Some studies observe that poor corporate governance caused the 2008 financial crisis, for example, lax board oversight encouraged excessive risk-taking (Erkens et al., 2012; Kowalewski, 2016). The role of the audit committee in overseeing risk-taking became increasingly critical after the financial crisis of 2008. According to a survey conducted by KPMG 2009, most members of the audit committee agreed that they had “*increased their hands-on involvement with management because of the financial crisis…*”, suggesting that they were required to improve the company's risk management by strengthening their oversight role in the period of the financial crisis. The Basel regulatory reforms pressured the banks' boards of directors to become involved with risk-management oversight and recommended a stand-alone risk committee as part of a board that would focus specifically on risk.

In addition, although some prior studies provide evidence that the audit committee played an important role in overseeing risk-taking and preserving bank stability (Sun and Liu, 2014; Nguyen and Dang, 2020), they focused only on conventional banks. Some studies recently found many differences between the risk-taking of conventional banks and that of Islamic banks. In contrast to the failures of conventional banks, Islamic banks performed effectively and stably since the financial crisis (Chapra, 2011; Green, 2010). Actually, charging interest payments are prohibited in Islamic banks. They must apply the risk-sharing model; thus, Islamic banks are better than conventional at managing risks (Hassan et al., 2019). Bilgin et al. (2021) provide evidence that credit growth is weakly affected by economic uncertainty in Islamic banks but strongly in conventional banks, implying that conventional banks are less stable than Islamic banks.

Moreover, many studies show that corporate governance differs widely between conventional and Islamic banks. While corporate governance of conventional banks comprises the board of directors and its committees, referred to as “single-layer”, the institution of Sharia committees in Islamic banks makes their governance “multi-layer” (see Mollah and Zaman, 2015). The difference is rooted in the fact that the Islamic perspective sees the practice of corporate governance as a Muslim's obligation to God, thus leading to the existence of, and obedience to, the “implicit” contract with God and the “explicit” contract with humans. In the end, these place God and Islam itself in roles as key players in the practice of corporate governance. This contrasts with the conventional point of view that focuses on the material aspects of governance. In practice, the differences are minor. The mechanism and tools for the effective implementation of corporate governance are relatively similar. Nevertheless, as Islamic financial institutions deal with more complicated financial transactions and must comply with Sharia rules, they require relatively stronger internal controls. Islamic banks establish a Sharia committee to review and ensure that Sharia-compliant products are in line with Sharia rules. The ethics committees and religious beliefs in Islamic banks helps the boards avoid poor-quality lending and take less risk. Overall, the corporate governance of Islamic banks, with Sharia supervision as an additional mechanism, suggests that Islamic banks showed less financial difficulty than that faced by conventional banks (see Mollah and Zaman, 2015). Analyzing banks in Gulf Cooperation Council (GCC) countries, Raouf and Ahmed (2020) provide evidence that risk governance, i.e corporate governance related to risk management, in Islamic banks is less effective than in conventional banks. Therefore, as an additional mechanism, the Sharia committee in Islamic banks may play an important role in oversight risk-taking.

Although the roles of the audit committee and the Sharia committee become more and more important in banking sectors, there is a lack of empirical data evaluating the role of Sharia and audit committees in oversight risk-taking in both Sharia and conventional banks. Although Mollah et al. (2017) provide strong evidence that Islamic banks with effective corporate governance allows them to take higher risks and achieve better performance than conventional banks overall, which of the factors that make a difference between the kinds of banks were not investigated. In fact, not all components of the governance structure of banks play a role in oversight risk-taking. By focusing on the roles of the audit committee and Sharia committee, our studies can help both conventional and Islamic banks to enhance the effectiveness of corporate governance and reduce risk-taking behavior in appropriate ways.

Our study examines whether the “multi-layer” mechanism of governance in Islamic banks and the “single-layer” mechanism of governance in conventional banks can reduce the banks' risk-taking. Specifically, we examine the relationship between Sharia and audit committees’ effectiveness and risk-taking behaviors in Islamic banks vis-à-vis conventional banks. Our focus on this comparison is important because there has been some debate about profitability, efficiency, and stability that casts doubt on the current state of Islamic banks (see Ariss, 2010; Beck et al., 2013; Abedifar et al., 2013; Bourkhis and Nabi, 2013; Hasan and Dridi, 2010). This study contributes to the prior studies in some ways.

First, this is the first study to examine the impact of audit committee's effectiveness on risk-taking in both conventional and Islamic banks, as well as the relationship between the Sharia committee's effectiveness and risk-taking in Islamic banks. Sun and Liu (2014) find that conventional banks with audit committees whose members have long board tenure have a lower risk but busy directors increase bank risk. They suggest that audit committees have oversight in risk-taking in conventional banks. Additionally, we examine the audit committee's role of performing oversight of risk-taking in both conventional and Islamic banks. Due to the differences between corporate governance of Islamic and conventional banks, the role of the audit committee may differ. Our result shows that only the audit committee's effectiveness can reduce the risk-taking in conventional banks. Similarly, the effectiveness of the Sharia committee can reduce risk-taking in Islamic banks.

Second, we examined the effectiveness of the audit and Sharia committees on the banks' risk-taking behavior during the 2008 financial crisis. This enabled us to examine whether the roles of corporate governance only manifest itself in exceptional times, which was not examined in past literature. Thus, we extended the literature (e.g., Sun and Liu, 2014; Pathan, 2009) in terms of the different effects of audit and Sharia committees effectiveness on banks’ risk-taking during different time periods by splitting the sample into multiple periods. We find that the oversight risk-taking role of audit and Sharia committees in conventional and Islamic banks respectively remained unchanged in all periods. However, the role of the audit committee in Islamic banks was found important in crisis period.

Finally, we examine what mechanism compels the audit and Sharia committees' effectiveness in constraining banks' risk-taking. Our results suggest that audit and Sharia committees’ effectiveness principally drives bank risk lower via different means: the audit committees of conventional banks drive bank risk lower via incentives to maintain higher capital ratios as well as through the reallocation effect for profit, but the Sharia committees drive bank risk lower via incentives to increase efficiency and reduce the volatility of profits in Islamic banks.

This article proceeds as follows: Section 2 outlines relevant literature and develops hypotheses about the association of audit committees, Sharia committee effectiveness with risk-taking behavior. Section 3 explains the methodology used in empirical tests and describes the data set. Section 4 contains the main results and Section 5 is conclusion.

## 2 Literature review and hypothesis development

### 2.1 Audit committee effectiveness and bank risk-taking

According to the moral hazard theory, shareholders encourage the bank management to invest in high-risk projects. Galai and Masulis (1976) state that the shareholders effectively hold a “call option” on the firm's value with an exercise price of the total amount of debt outstanding. With a risk-insensitive deposit insurance premium, bank shareholders want to increase leverage and bank risk. Therefore, they encourage the bank management to invest in “excessively” risky projects that potentially benefit shareholders at the expense of the deposit insurance fund and the taxpayers who back it. Pathan (2009) supports this view and finds that strong boards (i.e., boards that reflect shareholders' interests) positively affects banks' risk-taking. However, Boards has the role in oversight risk as a requirement of the Basel Committee. Akbar et al. (2017) and Younas et al. (2019) find that Board independence has a negative relation to risk-taking, and Board effectiveness enables better monitoring of the CEO, which leads to decision-making based on a more appropriate level of risk. In conventional banks, the Board usually plays the oversight risk-taking role through the audit committee (Sun and Liu, 2014). Nguyen (2021) provide evidence that audit committee can enhance bank stability. Therefore, the high effectiveness of an audit committee can reduce the bank's risk-taking.

In addition, per agency theory, the value of managers is mainly concentrated in the companies they manage, aside from when shareholders' investments are distributed in a diversified way. The managers tend to avoid risky strategies to protect their jobs. Through their oversight role, the audit committee can support bank managers in avoiding excessive risk-taking. Since the audit committee may constrain the bank's risk-taking, we develop the following hypothesis:

H1a The audit committee effectiveness negatively associates with risk-taking in conventional banks.

Given the differences in corporate governance between conventional banks and Islamic banks, the main function of the audit committee at an Islamic bank is to review and supervise the financial reporting, as well as provide oversight of the internal and external auditors (Safieddine, 2009). Thus, they may not have an oversight risk function. Based on five dimensions of risk governance (Board, Risk committee, Audit committee, Chief risk officer and Internal audit), Raouf and Ahmed (2020) report the lower strength of risk governance in Islamic banks than conventional banks. It implies that risk governance in Islamic banks has lower effectiveness in oversight risk-taking than conventional banks. In addition, Haddad et al. (2021) report the positive relationship between audit committee and banks’ liquidity in conventional banks, but this relation is unclear in Islamic banks. Therefore, we expect that the audit committees in Islamic banks have no role in oversight risk-taking, and we propose the null hypothesis:

H1b There is no relationship between the audit committee effectiveness and Islamic bank's risk-taking.

### 2.2 Sharia committee's effectiveness and risk-taking in Islamic banks

Unlike conventional banks, Islamic banks are responsible for ensuring compliance with the Sharia rules of their operations, products, instruments, management, and practices. In this paper, we argue that the Sharia committee has an oversight role in risk-taking, as well as the responsibility to reduce the moral hazard problem in Islamic banks. Some past studies support this view. Hamza (2013) finds that non-compliance with Sharia affects public confidence in Islamic finance and exposes Islamic banks to incredible risk. Furthermore, based on Islamic rules, Islamic banks have a higher asset quality, higher intermediation ratio, more stability, and better capitalization (Beck and Demirguc-Kunt, 2013). Safieddine (2009) finds that the key roles and responsibilities of the Sharia committee typically include setting Sharia-related rules and overseeing compliance; advising the boards of directors; and issuing verdicts (fatwa) to enable confidence with respect to Sharia compliance. These roles of the Sharia committee make Islamic banks more stable and less risky than conventional banks, as some previous studies concluded (El-Hawary et al., 2007; Hasan and Dridi, 2011). Elamer et al. (2020) also find that Sharia supervisory boards can increase the operational risk disclosures of Islamic banks in MENA countries. This finding indicates that Sharia supervisory boards play an important role in the oversight of operational risk. Moreover, Sharia rules prohibit management from taking excessive risks for short term profit when such behavior does not truly maximize bank value. AlAbbad et al. (2019) finds that the size of the Sharia supervision board and the proportion of busy board members on Sharia supervision boards positively and significantly influence Islamic banks' asset-return and insolvency risks. This indicates that the Sharia supervision board's quality affects bank risk by reviewing Sharia compliance. Overall, we expected that the Sharia committee could constrain risk-taking, and as such our second hypothesis is:

H2 Sharia committees' effectiveness is negatively correlated with bank risk-taking in Islamic banks.

## 3 Data description and methodology

### 3.1 Data description

This paper uses data from Bankscope (Orbis Bank Focus), a global database with data on both listed and non-listed banks for the period 2002–2018. Some variables are collected manually from banks’ financial statements, annual reports, and bank websites. For the purposes of this study, we use a sample that comprises the 10 countries with the large number of Islamic banks. These countries have both conventional and Islamic banks, which allows us to control for any unobserved time-variant effect by introducing country-year dummy variables. We initially selected all the banks in each country from which annual reports were published and which had enough information about audit and Sharia committees over all the periods covered by this study (i.e., pre-crisis, crisis, and post-crisis). We then excluded the banks that lacked data. Our final data includes 159 banks, of which 57 are Islamic banks. We also eliminated outliers in all variables by winsorizing at the 1st and 99th percentiles within each country. Our data includes 2,028 observations after excluding outliers as well as observations with missing data. The sample distribution is presented in Table 1.

**Table 1:** Sample distribution.

Country	Conventional banks	Islamic banks	Full sample	Obs	Percentage
Bahrain	11	13	24	365	18%
Indonesia	15	4	19	218	11%
Malaysia	20	12	32	468	23%
Pakistan	18	8	26	315	16%
Singapore	8	2	10	125	6%
Kuwait	4	5	9	118	6%
Qatar	5	3	8	93	5%
Saudi Arabia	7	2	9	84	4%
UAE	5	4	9	75	4%
Bangladesh	9	4	13	167	8%
Total	102	57	159	2028	100%

Note: The study used data of 159 banks (57 Islamic banks and 102 conventional banks) in 10 countries from 2002 to 2018.

### 3.2 Research methodology

#### 3.2.1 Measures of bank risk-taking

Our primary measure of bank risk-taking is the Z-score of each bank which was used in the literature (Pathan, 2009; Berger et al., 2016; Nguyen, 2020). The Z-score captures the number of standard deviations by which returns must diminish in order to deplete the equity of a bank. The higher the Z-score value, the lower the bank's risk-taking. We calculate Z-score as follow:

(1) $$\text{Z}=\frac{\text{ROA}+\text{ETA}}{\text{δROA}}$$

where ROA is the ratio of return on assets and δROA is its standard deviation. ETA is the ratio of equity on assets.

To check the robustness of the results, this study also uses some alternative measures of bank stability. First, we use the natural logarithm of the Z-score, which has also been used in previous studies (Aljughaiman and Salama, 2019; Houston et al., 2010). Second, we use the proportion of non-performing loans to total loans (NPLS) as a dependent variable. NPLS is another measure of bank risk-taking which is used in the literature (Dwumfour, 2017; Jiang et al., 2020). The lower the ratio, the lower the bank risk-taking. Finally, we use Altman's Z-score (see Altman, 1968) as an alternative bank risk-taking measure. The higher the Altman's Z-score, the lower the risk-taking and the lower the odds that a bank is heading for bankruptcy.

#### 3.2.2 Measures of audit committee and Sharia committee effectiveness

We measure the effectiveness of the audit and Sharia committees in multiple dimensions. We consider the following five characteristics in the audit and Sharia committees as a proxy for their effectiveness. We used these proxies in our model to test our hypothesis.

(1) Female members: Eckel and Grossman (2008) posit that women are more risk-averse in decision-making. Barber and Odean (2001) and Niederle and Vesterlund (2007) consider women less overconfident and more sensitive to risk. They also manage risk-taking better than their male counterparts. In addition, some prior studies provide evidence that women are more effective in their oversight role. Bennouri et al. (2018) postulate that female directors possess high monitoring capabilities and contribute to the board's human capital more than their male counterparts. Chen et al. (2018) also contend that firms with female directors tend to invest more in innovation to make their firms more efficient. Based on prior studies, we expect that a large number of female members on a committee will result in a more effective audit and Sharia committee. Therefore, our first measure of committee effectiveness is the percentage of female members on the committee.
(2) Audit committee and Sharia committee size: Evidence from prior studies suggests that a large board will create a more complex and less effective internal corporate governance (Boone et al., 2007; Mak and Kusnadi, 2005; Mollah and Zaman, 2015). Several studies that support this viewpoint find that a larger board will reduce firm performance (Guest, 2009; Kao et al., 2019). Based on these studies, we might expect that smaller audit and Sharia committees may oversee risk-taking more effectively. We, therefore, use the committee's size which is measured by the number of members on the committee as a second measure of the audit and Sharia committees' effectiveness.
(3) Audit committee and Sharia committee independence: Many studies agree that the presence of independent directors can increase the audit committee's effectiveness. For example, Alderman and Jollineau (2020) contend that an audit committee's independence is positively associated with the autonomy of the auditor, thus increasing the effectiveness of audit works. Raimo et al. (2021) maintain that an audit committee's independence can enhance the quality of financial reporting. In addition, Mohamad and Muhamad Sori (2016) also find that the independence of the Sharia committee leads to more efficient Sharia decisions. Based on previous studies, we believe that the independence of the audit and Sharia committees may make their oversight roles more effective. Following Xie et al. (2003), our third measure of audit and Sharia committee effectiveness is the ratio of outside directors to total members.
(4) Accounting or financial expertise: From a risk management perspective, directors with accounting or financial knowledge and experience are better at risk assessment. Therefore, the proportion of accounting or financial experts on boards and committees can improve risk monitoring. García-Sánchez et al. (2017) assert that financial experts on audit committees can reduce insolvency risk and support the monitoring advantage hypothesis of financial expertise. Moreover, accounting and financial experts on boards and committees enhanced internal control quality (Agrawal and Chadha, 2005; Krishnan, 2005). Therefore, we expect that accounting or financial experts enable audit committees, as well as Sharia committees, to be more effective in overseeing managers' risk-taking. Following DeFond et al. (2005), accounting or financial expertise is people who have experience as accountants, auditors, or financiers or have an accounting or finance degree. Therefore, we use the proportion of accounting or financial expertise as the fourth measure of audit and Sharia committee effectiveness.
(5) Meeting frequency: Conger et al. (1998) suggest that more frequent board meetings improve a board's effectiveness, as the sessions are a crucial dimension of board operations. Moreover, firms with a higher number of audit committee meetings have less financial restatement (Abbott et al., 2004) and are associated with lower earnings management (Xie et al., 2003). These studies suggest that the committees that meet regularly during the financial year are linked to effective monitoring. Basiruddin and Ahmed (2019) also find that a higher frequency of Sharia committee meetings reduces the risk of Sharia non-compliance. Therefore, frequently meetings may suggest that the committee is hard-working and may make them more effective in their oversight role. Our fifth measure of audit and Sharia committee effectiveness is the number of meetings per year of the audit committee and Sharia committee.

#### 3.2.3 Other control variables

At the bank level, we control bank size by using the natural logarithm of the total assets. In addition, we use a diversification index to control diversification (Laeven and Levine, 2009; Shim, 2019). We also control asset quality by using the loan loss provisions to the ratio of the total assets. Banks that have been selected for IPOs usually have more efficiency than others (Zhang et al., 2014). We, therefore, control IPO by using a dummy variable. The dummy variable equals 1 if the bank is listed in the year of observation and 0 otherwise. As a result, listed banks are expected to have a lower risk than others. In addition, we control the effect of banking spread concerning their “traditional activities” in bank risk-taking by using NIM (net interest margin). The higher values of NIM are expected to reduce risk-taking.

At the country level, we control for differences in economics across countries by using several country-level variables to. Prior studies provide evidence that the macro-economic environment can affect bank risk-taking (Moudud-Ul-Huq, 2019; Zhang et al., 2021). First, we control the country economic development by using the natural logarithm of the GDP per capita. Second, we include the CR3 ratio to control the level of bank competition (Chong et al., 2013). Finally, we include the Worldwide Governance Indicators to proxy for institutional quality indexes (Kaufmann et al., 2006). These indicators are constructed from 276 individual variables taken from 31 sources produced by 25 organizations. Table 2 show all variables which used in this study.

**Table 2:** Definitions of variables.

Variables	Measure
**Panel A: Bank risk-taking (BRT)**	
Z-score	Z-score = [Return on assets ratio + (Equity on Total assets ratio)]/δ(Return on assets ratio)
LZ-score	Natural logarithm of the Z-score
AZ-score	$\text{AZ}-\text{score}=1.2{\text{x}}_{1}+1.4{\text{x}}_{2}+3.3{\text{x}}_{3}+0.6{\text{x}}_{4}+1.0{\text{x}}_{5}$, “where x1 is the working capital/total assets, x2 the retained earnings/total assets, x3 the earnings before interest and taxes/total assets, x4 the market value of equity/total assets, and x5 the sales/total assets”
NPLS	Nonperforming loans to total loans ratio
**Panel B: Sharia and audit committees' effectiveness**	
1. Female members on audit committee/Sharia committee (AFM-SFM)	Proportion of female members on the audit/Sharia committee
2. Audit committee/Sharia committee size (ACS-SCS)	Number of audit/Sharia committee members
3. Audit committee/Sharia committee independence (ACI-SCI)	Proportion of independent directors on the audit/Sharia committee
4. Accounting or financial expertise in audit committee/Sharia committee (AFE-SFE)	Proportion of accounting or financial expert on the audit/Sharia committee.
5. Meeting frequency in audit committee/Sharia committee (AMF-SMF)	Number of meetings of the audit/Sharia committee a year
**Panel C: Other control variables**	
Diversification index (DIV)	A diversification index - It is defined as: 1 − $\left|\frac{Net\;interest\;income-Other\;operating\;income}{Total\;operating\;income}\right|$
Bank size (BSIZE)	Log (total assets)
Net interest margin (NIM)	NIM ratio
Listed bank (IPO)	Dummy variable that equals one for listed banks; otherwise zero
Asset quality (ASSQ)	Ratio of loan loss provisions to total assets
Bank concentration (CR3)	Concentration ratio for three largest banks = $\overset{3}{\underset{n=1}{\sum }}\text{branchn}/\overset{ki}{\underset{k=1}{\sum }}\text{branchk}$, n = 1; . . .; 3 are the three largest banks by number of bank branches
GDP per capita (GDP)	Natural logarithm of GDP per capita in a year
Institutional quality (INS)	Quality of Governance Index – mean value of six governance dimensions for each country every year

### 3.3 Empirical models

To test our hypotheses, we use the following model

(2) $$BRT_{it}=\alpha_{0}+\alpha_{j}\sum_{j=1}^{5}ADC_{it}+\beta_{k}\sum_{k=1}^{5}SRC_{it}+\gamma_{1}DIV_{it}+\gamma_{2}BSIZE_{it}+\gamma_{3}NIM_{it}+\gamma_{4}IPO_{it}+\gamma_{5}ASSQ_{it}+\gamma_{5}CR3_{t}+\gamma_{6}GDP_{t}+\gamma_{7}INS_{t}+\gamma_{i}+\gamma_{t}+\gamma_{c}+\varepsilon_{it}$$

where ADC is a matrix of audit committee characteristic variables, SRC is a matrix of Sharia committee characteristic variables, α, β andγ are the parameters to be estimated, $\gamma_{i}$, $\gamma_{t}$, and are the bank, year, and country fixed effect respectively, ε is the error term. All variables are summarized in Table 2.

### 3.4 Estimation method

Fixed effect and random effect estimation methods are widely used for panel data in the literature. In this study, the primary estimation method for Eq. (2) is the fixed effect (FE) technique after performing the Hausman Test (Wooldridge, 2002).

## 4 Empirical analysis

### 4.1 Descriptive statistics and correlation matrix

The overall descriptive statistics of the main variables are presented in Table 3. We report the descriptive statistics in columns 3–9. We present the mean for Islamic banks, the mean for conventional banks, and the two-sample t-test (comparison of the means of Islamic banks vs. conventional banks) in columns 10–12.

**Table 3:** Descriptive statistics.

Full sample									Ibs sample mean	CBs sample mean	Two-sample T-test
Variables	Obs	Mean	Stdev	Min	Q_25	Q_50	Q_75	Max			
(1)	(2)	(3)	(4)	(5)	(6)	(7)	(8)	(9)	(10)	(11)	(12)
Z-score	2028	7.942	5.278	-4.732	2.224	4.275	8.562	23.823	9.234	8.125	3.916∗∗∗
LZ-score	1963	3.152	0.157	-2.563	-1.154	1.658	3.652	5.641	3.214	2.925	2.235∗∗
AZ-score	2028	8.923	7.215	-93.651	-20.325	17.269	52.123	72.168	9.236	6.122	0.423
NPLS	2028	0.045	0.081	0.000	0.002	0.028	0.031	0.581	0.036	0.049	-8.922∗∗∗
AFM	2028	0.312	0.445	0	0	0	0.225	1	0.285	0.405	-3.924∗∗
ACS	2028	0.143	0.345	0	0	0	0.214	1	0.167	0.135	0.224
ACI	2028	0.785	0.372	0	1	1	1	1	0.686	0.793	-0.287
AFE	2028	0.689	0.296	0	0	0	1	1	0.587	0.703	-11.265∗∗
AMF	2028	5.216	0.456	2	1.246	4.445	6.269	13	4.926	5.412	-0.228
SFM	2028	0.412	0.341	0	0	0	0.225	1	-	-	-
SCS	2028	0.241	0.326	0	0	0	0.214	1	-	-	-
SCI	2028	0.683	0.271	0	0	1	1	1	-	-	-
SFE	2028	0.619	0.216	0	0	0	1	1	-	-	-
SMF	2028	6.211	0.516	3	1.246	4.445	6.269	16	-	-	-
DIV	2028	0.434	1.561	-22.135	0.282	0.458	0.674	1	0.531	0.411	0.248
BSIZE	2028	9.702	0.698	6.257	9.175	9.794	10.821	12.606	8.726	11.743	-4.665∗∗∗
NIM	2028	0.083	0.044	-0.013	0.023	0.039	0.147	0.325	0.075	0.091	-0.541
IPO	2028	0.218	0.427	0	0	0	1	1	0.128	0.319	-1.289∗
ASSQ	2028	0.007	0.027	-0.026	0.001	0.005	0.012	1.574	0.006	0.007	-0.125
CR3	2028	0.672	0.145	0.248	0.356	0.522	0.69	0.97	-	-	-
GDP	2028	4.215	0.316	2.84	3.023	3.481	3.634	5.82	-	-	-
INS	2028	-0.421	0.435	-1.565	-0.512	-0.387	-0.318	1.655	-	-	-

This table presents the descriptive statistics for all variables. IBs and CBs are Islamic and conventional banks, respectively. See Table 2 for variable definitions. ∗ Significance at the 1% level, ∗∗ Significance at the 5% level, ∗∗∗ Significance at the 10% level.

First, we find that for the Islamic banks (IB) sample (conventional banks-CB sample; full sample), the mean Z-score is 9.234 (8.125; 7.942). T-tests reveal a significant difference in bank risk-taking between conventional banks and Islamic banks. We find the same result for the LZ-score and the NPLS. The means of the audit committee characteristic variables for the IB sample (CB sample; full sample) are: the proportion of female members (AFM) is 0.285 (0.405; 0.312); audit committee size (ACS) is 0.167 (0.135; 0.143); audit committee independence (ACI) is 0.686 (0.793; 0.785); the proportion of financial experts (AFE) is 0.587 (0.703; 0.689); and meeting frequency of the audit committee (AMF) is 4.926 (5.412; 5.216). The results of the t-tests in column 12 show a significant difference between Islamic banks and conventional banks only for the AFM and AFE.

We find that the means of the Sharia committee characteristic variables are: the proportion of female members (SFM) is 0.412; Sharia committee size (SCS) is 0.241; Sharia committee independence (SCI) is 0.683; the proportion of financial experts (SFE) is 0.619; and meeting frequency of the Sharia committee (SMF) is 6.211. In addition, the means of the bank-specific variables for the IBs (CBs; full sample) are: diversification index (DIV) is 0.531 (0.411; 0.434); bank size (BSIZE) is 8.726 (11.743; 10.702); net interest margin (NIM) is 0.075 (0.091; 0.083); listed bank (IPO) is 0.128 (0.319; 0.218); and asset quality (ASSQ) is 0.006 (0.007; 0.007). The results of the t-tests in column 12 show a significant difference between the IBs and CBs in bank size (BSIZE) and listed bank (IPO). The country-specific variables have the following means: bank concentration (CR3) is 0.672; GDP per capita (GDP) is 4.215; and institutional quality (INS) is -0.421.

To examine the correlation among variables, we present the Pearson's pairwise correlation coefficients in Table 4. The results show that the maximum value is 0.728 for a positive correlation between institutional quality (INS) and GDP per capita (GDP). All other coefficients are lower than 0.7, indicating that problem of multicollinearity may be not a concern. However, the correlation measures might be highly unreliable indicators of the relationships among many variables such as bank size and ownership structure, and other attributes are likely to affect bank stability. Therefore, we continued to test our hypotheses by using a multiple regression framework.

**Table 4:** Correlation matrix.

	Z-score	AFM	ACS	ACI	AFE	AMF	SFM	SCS	SCI	SFE	SMF	DIV	BSIZE	NIM	IPO	ASSQ	CR3	GDP	INS
Z-score	1.000																		
AFM	0.183	1.000																	
ACS	-0.246∗∗	0.423	1.000																
ACI	0.311∗	0.075∗	0.476	1.000															
AFE	-0.231	-0.091	-0.293∗∗	-0.173	1.000														
AMF	0.103∗	-0.112∗∗	-0.393	-0.044	-0.282∗∗	1.000													
SFM	0.026∗∗	0.387	0.025	0.338∗∗∗	0.153∗∗	0.113∗	1.000												
SCS	-0.272∗∗∗	0.072	0.187∗∗∗	0.098	-0.224	-0.009∗	0.042∗∗	1.000											
SCI	-0.134∗	0.234	0.425	0.154	-0.385∗	0.252∗∗	0.027	0.252∗	1.000										
SFE	0.189	0.352∗	0.152∗∗	-0.019∗	-0.162∗∗∗	-0.301	0.220∗∗∗	0.017	-0.154∗∗	1.000									
SMF	0.429∗∗	0.298	0.324	0.392∗∗∗	0.004∗∗	0.091∗∗∗	0.161∗∗	0.021∗∗	-0.001∗	0.017	1.000								
DIV	-0.371	-0.173∗	-0.123∗	-0.595	0.025	0.013∗	-0.311∗∗	0.243∗∗∗	0.318∗	-0.183∗∗	-0.005∗	1.000							
BSIZE	-0.175	-0.274	-0.004	-0.335∗∗	0.165∗	-0.064	-0.232	-0.078∗∗	-0.228∗∗	0.006∗∗	-0.142	0.056	1.000						
NIM	0.213∗∗	0.117∗∗∗	0.136	-0.106	-0.014∗∗	-0.032	0.006∗	0.331	0.318∗∗∗	0.271∗	0.133∗∗∗	-0.032	-0.212	1.000					
IPO	0.092∗	0.184	0.204∗∗	0.013∗∗∗	-0.183	0.151∗	0.015∗∗	0.338∗∗	0.080	-0.352∗∗∗	0.027∗	0.031	-0.241∗∗	-0.012∗	1.000				
ASSQ	0.257	0.327∗∗	0.027	-0.211	0.451∗∗	-0.371	-0.361	0.274	0.124∗∗	0.142∗	0.056∗∗	0.148∗∗	0.018	0.235∗∗	-0.122	1.000			
CR3	0.021∗∗∗	0.565	0.041∗	0.121∗	-0.210	-0.373∗∗	0.068∗∗	0.403∗∗∗	0.004∗	0.524	0.006	-0.124∗	-0. 041∗	0.145	-0.007∗∗	0.056∗∗	1.000		
GDP	0.154∗∗∗	-0.159	-0.352∗∗	0.001	0.381∗∗	-0.109	-0.008∗	0.249	0.182∗∗	-0.246∗∗∗	0.047∗∗	0.021∗∗	0.174∗∗	0.031∗∗∗	0.137∗	0.043∗	-0.170∗∗	1.000	
INS	0.065∗	0.359	0.066	-0.153	-0.123	-0.262∗	0.174	-0.007∗∗	0.039∗	0.416	0.024	-0.071	-0.104	0.007	-0.062	0.189	0.372∗∗∗	0.728∗∗	1.000

Note: ∗ Significance at the 1% level, ∗∗ Significance at the 5% level, ∗∗∗ Significance at the 10% level.

### 4.2 Effects of audit and Sharia committee effectiveness on bank risk-taking

We use fixed effect (FE) estimation and present the results of our analysis of the relationship among audit committee structure, Sharia committee structure variables, and bank risk-taking in Tables 5 and 6. In Table 5, we present the regression results of Eq. (2) examining the effect of the audit committees' and Sharia committees' effectiveness on bank risk-taking over the period (2002–2018). Model (1) is for the Islamic bank sample, model (2) is for the conventional bank sample, and model (3) is for the full sample. First, we find that audit committee independence, accounting or financial expertise in the audit committee, and meeting frequency of the audit committee all have a positive relationship to the Z-score in both the conventional bank sample and full sample. The results are quite consistent with prior studies (Sun and Liu, 2014; Nguyen, 2021; Xie et al., 2003). The empirical results of Liu and Sun (2021) show that audit committee members' independence and expertise can constrain bank risk-taking behavior and enhance performance. Our results indicate that the audit committee's effectiveness may constraint a bank's risk-taking, and an appropriate audit committee structure can enhance its effectiveness in oversight risk-taking in conventional banks. This result strongly supports the H1a hypothesis and provides strong evidence that audit committee in conventional banks plays an important role in oversight risk-taking. However, we find no evidence of a relationship between the effectiveness of audit committee and risk-taking in Islamic banks. We find only a positive relationship between AMF and the Z-score, with significance at 10% level. It is weak to reject the H1b hypothesis. This result is consistent with some literature findings that audit committee may not be a key factor of corporate governance in Islamic banks. For example, the empirical results of Alkdai and Hanefah (2012) show that audit committee size and financial experts on an audit committee have relation to Malaysian firm's earning management. Budiyono and Sabilla (2021) find that audit committees in Indonesian Islamic banks do not affect financial reporting quality. Moreover, risk governance in Islamic banks has lower effectiveness than conventional banks (Raouf and Ahmed, 2020); thus audit committee may not consider constraint risk-taking effectively in Islamic banks. Second, we find that female members on the Sharia committee and accounting or financial expertise on the Sharia committee positively relate to the Z-score, but that Sharia committee size negatively affects the Z-score (see models 4 and 5). This strongly supports the H2 hypothesis and is consistent with previous studies (Gul et al., 2011; Mollah and Zaman, 2015). However, the results in Table 5 do not provide evidence about the relationship between the independence of the sharia committee and risk-taking. This finding does not support prior studies which found that independence can enhance Sharia committee effectiveness (Hamza, 2013; Basiruddin and Ahmed, 2019). Although not all Sharia committee variables affect risk-taking, we have evidence that Sharia committee effectiveness can increase or influence constraint bank risk-taking in Islamic banks. Overall, audit committees have a role in the oversight of risk-taking in conventional banks, but they do not have the same role in Islamic banks. In Islamic banks, Sharia committees play a crucial role in the oversight of risk-taking.

**Table 5:** Audit committee and Sharia committee effectiveness on bank risk-taking, all periods (2002–2018).

Variable	Audit committee effectiveness			Sharia committee effectiveness	
	(1) IBs	(2) CBs	(3) Full sample	(4)	(5)
AFM	0.242∗	-0.235	-0.423		0.535
ACS	-0.031	-0.261	0.225		-1.223
ACI	-0.143	0.203∗∗∗	0.115∗∗		0.562
AFE	0.312	0.426∗∗∗	0.216∗		0.445∗
AMF	0.281	0.351∗∗∗	0.237∗∗		0.521
SFM				0.382∗∗∗	0.641∗∗∗
SCS				-0.912∗∗∗	-1.833∗∗∗
SCI				0.312	1.509
SFE				0.525∗∗∗	0.673∗∗
SMF				0.452	3.248
DIV	1.821∗∗	3.158∗	3.211	2.269	1.924∗
BSIZE	-0.323∗∗	-0.213∗∗	-0.457∗∗	-1.822∗	-1.223∗∗
NIM	0.592∗∗	0.241∗	1.326∗∗∗	2.521∗∗	1.137∗
IPO	0.137	-5.152	-3.642	3.653	-2.621
ASSQ	-5.622	-3.126	-2.125	-1.119	-3.174
CR3	3.215∗∗	5.396∗∗∗	6.234∗∗∗	3.278∗∗∗	4.100∗∗∗
GDP	1.526	-5.513	-2.265	-7.368	-2.281
INS	0.264∗∗	2.294∗∗∗	1.497∗∗∗	2.325∗∗	3.156∗
R2	0.41	0.38	0.35	0.37	0.44
Year fixed effect	yes	yes	yes	yes	yes
Bank fix effect	yes	yes	yes	yes	yes
Country fixed effect	yes	yes	yes	yes	yes
Obs	895	1133	2028	895	895

Note: This table presents the Fixed effect estimation results for the effects of the audit committee's effectiveness, Sharia committee's effectiveness, and bank risk-taking for all periods. Model 1 to 3 shows the regression results of testing the effect of audit committee effectiveness on risk-taking for Islamic banks, conventional banks, and full data respectively while models 4 and 5 show the regression results of testing the effect of Sharia committee effectiveness on risk-taking. Model 4 does not include audit committee variables while model 5 includes both audit committee variables and Sharia committee variables. A description of the variables has been presented in Table 2. ∗ Significance is at the 1% level, ∗∗ Significance is at the 5% level, ∗∗∗ Significance is at the 10% level.

**Table 6:** Effect of audit committee and Sharia committee effectiveness on bank risk-taking: pre-crisis, crisis and post-crisis.

Variable	Pre-Crisis			Crisis			Post-Crisis		
	(1) IBs	(2) CBs	(3) Sharia committee	(4) IBs	(5) CBs	(6) Sharia committee	(7) IBs	(8) CBs	(9) Sharia committee
AFM	-0.352	-1.212	-4.186∗	-1.469	2.246	-1.421	-1.952	0.561	-0.372
ACS	0.321	-1.862	-2.239	-1.935	-3.287	3.849	4.245	-0.518	-2.281
ACI	1.387	1.762∗	0.763	0.281∗∗	0.995∗∗	1.232∗∗∗	1.643	1.7751∗∗∗	0.935
AFE	0.543	0.384∗∗∗	0.451	0.447	1.621∗∗	-0.653	1.318	0.221∗∗	1.903
AMF	-0.348	0.382∗∗	0.289	0.657	0.461∗∗	2.751	-1.464	0.681∗∗	0.675
SFM			0.322∗∗			1.246∗			0.698∗∗
SCS			-2.746∗∗∗			0.524∗∗∗			-2.790∗∗∗
SCI			-2.163			4.163			3.318
SFE			0.437∗∗			0.719∗∗			0.715∗∗∗
SMF			3.187			2.278			4.241
DIV	2.362∗∗∗	1.905∗∗	6.751	3.215∗∗	4.635∗	6.014	-2.458	1.668	2.928∗∗∗
BSIZE	-2.028∗∗	-0.724∗	-1.553∗∗	-1.092∗	0.363∗∗	-5.928	-3.391∗	1.765∗∗	-3.016∗
NIM	0.725∗	0.677∗∗	1.276∗	0.891∗	1.204∗	7.641	2.218	2.218∗	1.954∗
IPO	1.211	-9.165	-2.548	2.445	-1.129	-1.576	3.675∗∗	-1.167	-1.751
ASSQ	-3.092	-7.591	-3.194	-3.621	-3.535	8.135	-5.627	-2.915	-4.914
CR3	3.521∗	2.801∗∗∗	4.151∗∗	4.255∗	5.928∗∗∗	3.126∗	1.586∗	3.161∗∗∗	2.181∗∗
GDP	3.129∗	-3.702	-2.790	1.598	-8.512	4.715	3.572	-4.576	-7.613
INS	0.915∗	3.387∗∗∗	3.187∗	0.694∗∗	1.323∗∗∗	2.198	0.154∗	1.612∗∗∗	3.388∗
R^2^	0.34	0.37	0.37	0.35	0.34	0.36	0.34	0.39	0.40
Year fixed effect	yes	yes	yes	yes	yes	yes	yes	yes	yes
Bank fixed effect	yes	yes	yes	yes	yes	yes	yes	yes	yes
Country fixed effect	yes	yes	yes	yes	yes	yes	yes	yes	yes
Obs	320	340	320	171	304	171	404	489	404

Note: Table 6 presents the impacts of the audit committee's effectiveness, ​Sharia committee's effectiveness on bank risk-taking for pre-crisis, crisis, and post-crisis periods. Model 1, 4, and 7 show regression results of testing the effect of audit committee effectiveness on risk-taking in Islamic banks for all three phases of periods respectively. Model 2, 5, and 8 show regression results of testing the effect of audit committee effectiveness on risk-taking in conventional banks for all three phases of periods, respectively. Model 3, 6, and 9 show regression results of testing the effect of Sharia committee effectiveness on risk-taking in Islamic banks for all three phases of periods, respectively. ∗ Significance is at the 1% level, ∗∗ Significance is at the 5% level, ∗∗∗ Significance is at the 10% level.

Furthermore, we find that bank size has a negative association with the Z-score of both conventional and Islamic banks. Interestingly, both concentration and institutional quality have a positive significant association with the Z-score of conventional and Islamic banks, according to our findings. These results have indicated that the level of bank risk depends on the bank's net interest revenue, the level of competition, and the quality of institutions in the countries. Our findings are compatible with previous studies (Dwumfour, 2017; Houston et al., 2010; Beck et al., 2013; Klomp and de Haan, 2014). Because of the high degree of risk, this result suggests that major banks of both types should consider proper risk governance to restrict risk-taking.

As seen in Table 6, we further explore whether there is any change in audit and Sharia committees role in oversight risk-taking. Our sample was divided into three phases of periods (ie. pre-crisis, crisis, and post-crisis). We have defined the full period as 2002–2018, the pre-crisis period as 2002–2007, the crisis period as 2008–2011 (following the studies of Bennett et al., 2015; Arthur et al., 2015; and Andrieș and Ursu, 2016), and the post-crisis period as 2011–2018. Models 1–3 represent the pre-crisis phase of the period, models 4–6 represent the crisis phase of the period, and models 7–9 represent the post-crisis phase of the period.

The results in both the pre-crisis and post-crisis periods are consistent with our first result. These results are also consistent with Nguyen (2021). During the crisis period, however, we find that the audit committee independence (ACI) generally has a positive effect on the Z-score of both conventional and Islamic banks. However, coefficients on AFM, ACS, AFE and AMF are not significantly related to the Z-score. Thus, it is weak to reject hypothesis H1B. Interestingly, we find that the Sharia size (SCS) positively relate to the Z-score in the crisis period. This result may be explained by Baxter and Cotter (2009), who argue that large committees with members with varied expertise are more likely to have effective oversight. The sign of the coefficient of the Sharia committee's size, however, becomes negative in the post-crisis period.

### 4.3 Extensions

To better understand the driving forces behind the hypothesized mechanisms of the audit and Sharia committees' effectiveness, in this case measured by their efficiency with respect to bank risk, we performed two additional tests. First, we focused on the components of the Z-score to establish whether we could attribute the beneficial effect of the audit and Sharia committee variables on bank risk-taking to the effects of these variables’ capitalization level (E/A), profitability (ROA), or the volatility of profits (δROA).

In Table 7 we find that audit committee independence (ACI), accounting or financial expertise on the audit committee (AFE), and meeting frequency of the audit committee (AMF) are positively associated with efficiency (ROA) and capital ratio (E/A) in conventional banks. These findings indicate that the audit committee's effectiveness in conventional banks is largely due to constraints on banks' risk-taking via incentives to hold higher capital ratios as well as the reallocation effect of profits. These findings are consistent with Nguyen (2021). Aldamen et al. (2012) propose that smaller audit committees with more financial expertise can enhance both the market and accounting performance of a firm during a financial crisis. Kallamu and Saat (2015) find that independent audit committee members provide effective monitoring of the firm's management, thereby enhancing profitability and reducing the possibility of opportunistic behavior by management. We find, however, that the proportion of female members (SFM) and the proportion of financial and accounting experts (SFE) positively relate to ROA in Islamic banks (model 6) but negatively relate to δROA (model 9). The coefficient of SCS is negative and significant with the ROA but positive and significant with the δROA. This result indicates that the Sharia committee's effectiveness serves to constrain bank risk-taking via incentives to increase efficiency and reduce the volatility of profits in Islamic banks. Taken together, we find that the audit and Sharia committees' effectiveness have an effect on the bank risk of conventional and Islamic banks, respectively, but with a difference in transmission mechanism. Our findings provide an explanation about the mechanism – that corporate governance effectiveness influences risk-taking – which the literature does not sufficiently address.

**Table 7:** Effect of audit committee and Sharia committee effectiveness on the components of the Z-score.

Variable	E/A			ROA			δROA		
	(1) IBs	(2) CBs	(3) Sharia committee	(4) IBs	(5) CBs	(6) Sharia committee	(7) IBs	(8) CBs	(9) Sharia committee
AFM	5.412	-2.213	-5.143	3.614	1.622	-0.765	-2.434∗	1.182	-3.052
ACS	-12.315	-4.651	3.671∗	3.245	-5.190	2.927	2.623	2.422	-2.916
ACI	4.081∗	0.732∗∗	2.865	-0.383	0.521∗∗	4.174∗	2.518∗	-0.291	-0.539∗∗∗
AFE	0.161	2.541∗	0.613	3.120∗	1.625∗	-0.481	1.325	2.013	1.507
AMF	-0.639	0.923∗∗	1.254	2.187∗	0.081∗	3.281	-1.416	-0.562	-1.260
SFM			0.321∗			2.287∗∗			-0.612∗∗
SCS			-2.189			-1.065∗∗			3.265∗
SCI			-0.182			0.354			2.584
SFE			2.456			4.826∗∗∗			-0.675∗∗
SMF			2.898			3.871			4.241
DIV	2.764∗∗	2.347	-2.042	1.267∗∗	2.268∗∗	4.504	3.482	-3.614∗∗	3.921∗
BSIZE	-3.722	4.621∗	-3.258∗∗	1.0935	-2.946∗∗∗	-5.696∗	-2.421	2.358∗∗∗	-2.922∗
NIM	1.226	1.474∗∗	3.241	0.257	2.4781∗	-4.602	3.261∗∗∗	0.258∗	1.932∗
IPO	0.342	-4.260	-0.875∗∗	2.966∗	0.442	4.091	2.128∗	-4.180	-1.721
ASSQ	-3.853∗∗∗	-0.263	-3.821	-1.635	-1.944	2.865	-4.115	-3.141	-4.974
CR3	5.175	3.193∗∗	4.412∗	3.237∗∗	1.803∗∗	3.054∗∗	1.288	1.491∗∗∗	4.112∗∗
GDP	-2.354∗	-5.804	-3.225	4.156	3.025	3.255	5.401	-7.543	-2.908
INS	1.421∗∗	4.123∗∗∗	4.285∗	2.203∗	1.387∗∗	5.370∗∗	2.129	3.686∗∗∗	3.141∗
R2	0.26	0.31	0.33	0.31	0.34	0.34	0.32	0.29	0.35
Year fixed effect	yes	yes	yes	yes	yes	yes	yes	yes	yes
Bank fixed effect	yes	yes	yes	yes	yes	yes	yes	yes	yes
Country fixed effect	yes	yes	yes	yes	yes	yes	yes	yes	yes
Obs	895	1059	895	895	1059	895	895	1059	895

Note: this table presents the fixed effect estimation results for the effects of audit committee's effectiveness, Sharia committee's effectiveness on the components of the Z-score. Models (1), (4), (7) and (2), (5), and (8) examine the relationship of the audit committee's effectiveness and the components of the Z-score in Islamic banks and conventional banks, respectively. Models (3), (6), and (9) examine the relationship of the audit committee's effectiveness, Sharia committee's effectiveness, and the components of the Z-score in Islamic banks. See Table 2 for a description of the variables. ∗ Significance at the 1% level, ∗∗ Significance at the 5% level, ∗∗∗ Significance at the 10% level.

### 4.4 Robustness tests

In this section, we use an alternative measure of bank risk-taking and a two-step system GMM to ensure that the estimates are robust to unobserved heterogeneity, simultaneity, and dynamic endogeneity (if any). The two-step system GMM estimation method, which is introduced by Arellano and Bover (1995) and Blundell and Bond (1998), may be appropriate way to deal with endogeneity concerns associated with the various determinants of bank risk-taking. Moreover, by using Hansen's J-statistic, we test the instrument validity. To test for order serial autocorrelation, we apply the Arellano and Bond (1991). The effects of the audit and Sharia committee's effectiveness on some proxies of risk-taking are presented in Table 8.

**Table 8:** Effect of audit committee and Sharia committee effectiveness on bank risk-taking, GMM method.

Variable	Z-score			LZ-score			AZ-score			NPLS		
	(1) IBs	(2) CBs	(3) Sharia committee	(4) IBs	(5) CBs	(6) Sharia committee	(7) IBs	(8) CBs	(9) Sharia committee	(10) IBs	(11) CBs	(12) Sharia committee
AFM	5.418	0.419	-2.192	0.014	0.079	-3.102	-1.425	-2.673	1.283	0.008	0.019	-1.127
ACS	-2.312∗	-0.421	-1.585∗	0.125	-3.027	-1.025	3.042	-1.892	3.225∗	0.012∗	0.021	0.085∗
ACI	0.197	1.724∗∗	2.819	0.097	0.028∗∗	0.812	0.424	5.421∗∗∗	-3.841	0.007	-0.021∗∗	2.819
AFE	0.592	3.342∗∗∗	5.115	0.102∗	0.142∗∗∗	0.145	-4.815	1.305∗∗	2.165	0.002	-0.002∗∗	-3.196
AMF	-0.336	2.415∗∗	1.903	-0.006	0.419∗∗	1.043	3.312	4.449∗∗	0.952	-1.336	-0.015	0.915
SFM			2.484∗∗∗			0.074∗∗∗			3.929∗∗			-0.004∗∗∗
SCS			-0.922∗∗∗			-0.007∗∗∗			-6.516∗∗∗			0.192∗∗∗
SCI			3.451			0.469			0.873			2.441
SFE			3.783∗∗∗			0.713∗∗∗			1.242∗∗			-0.003∗∗∗
SMF			12.094			2.254			0.271			-2.15
DIV	3.719∗∗∗	5.823∗∗	2.917	1.029∗∗	1.203∗∗	4.912	8.219∗∗	4.715∗∗	0.422	-1.219∗	-1.723∗∗	0.847
BSIZE	-0.318∗∗∗	-1.685∗∗	-5.245∗	-0.117∗	-1.195∗∗	-0.242∗	-4.730∗∗∗	-5.454	-3.673	0.018∗∗	0.005∗∗	-1.215
NIM	2.217∗∗	0.284	0.275	3.207	0.981	1.225	3.574∗	4.623∗∗	2.248∗	3.217	0.784	0.075
IPO	-7.918	-6.168	-3.541	-1.910∗	-3.162	-1.561	1.342	-6.175	-2.507	-0.928	-2.1612	-4.201
ASSQ	-2.615	10.155	4.812	-5.411	8.154	-7.831	0.689	-5.476	-0.825	-2.615	1.251	3.810
CR3	4.285∗∗∗	3.467∗∗∗	2.173∗∗	0.215∗∗∗	0.468∗∗∗	1.203∗∗	-4.272	7.413∗∗∗	1.189∗∗	1.205	2.414∗∗∗	0.173∗∗
GDP	4.533∗∗	-7.515	-0.721	1.003∗∗	-5.541	-2.746	2.568∗	-2.548	-3.743	0.587∗∗	-0.524	-0.742
INS	2.145∗	2.316∗∗∗	4.281∗	0.124	0.312∗∗∗	4.221∗	3.672∗	1.359∗∗	3.881	-2.105∗	1.317∗∗∗	1.081∗
Hansen J test (p-value)	0.723	0.645	0.718	0.723	0.645	0.718	0.481	0.458	0.315	0.123	0.045	0.314
AR (1) (p-value)	0.000	0.000	0.002	0.012	0.018	0.009	0.001	0.023	0.012	0.054	0.016	0.013
AR (2) (p-value)	0.364	0.654	0.425	0.367	0.851	0.321	0.512	0.245	0.398	0.463	0.153	0.521
Obs	895	1133	895	868	1095	868	895	1133	895	895	1133	895

Note: This table presents the GMM estimation results for the effects of the audit and Sharia committee's effectiveness on bank risk-taking using Z-score (regression 1–3); LZ-score (regression 4–6); AZ-score (regression 7–9); and NPLS (regression 10–12), respectively. See Table 2 for a description of the variables. ∗ Significance at the 1% level, ∗∗ Significance at the 5% level, ∗∗∗ Significance at the 10% level.

We find that ACI, AFE, and AMF positively relate to the Z-score, LZ-score, and AZ-score and negatively relate to NPLS in conventional banks. The coefficient of the audit committee effectiveness variables is almost insignificant; SFM and SFE positively and SCS negatively relate to the Z-score, LZ-score, and AZ-score, and negatively relate to NPLS in Islamic banks. These results are consistent with our first finding and continue to support our hypothesis. The effectiveness of the audit and Sharia committees may constraint bank risk-taking.

The diagnostics tests in Table 8 show that the model is appropriate to test our hypotheses. The first-order autocorrelation (AR1) should be significant, and test results show a p-value lower than 0.05. The statistically insignificant AR2 (p > 0.05) in Table 8 suggests that if we fail to reject the null hypothesis that there is no serial correlation in the idiosyncratic disturbances. Likewise, the Hansen test results indicate that the instruments are valid in their respective estimation. Overall, the system GMM estimates in Table 8 demonstrate that the audit and Sharia committee's effectiveness are found to relate to bank risk-taking in a way consistent with our expectations.

## 5 Conclusion

Our research examines the effect of the effectiveness of the audit committee and Sharia committee on bank risk-taking and yields some important results. First, we find that the audit committee's effectiveness can oversee and constrain risk-taking in conventional banks but not in Islamic banks. The Sharia committee's effectiveness, however, can constrain risk-taking in Islamic banks. Second, the effectiveness of the audit committee and Sharia committee demonstrate different mechanisms that affect banks' risk-taking. Finally, the role of the audit committee in conventional banks and the role of the Sharia committee in Islamic banks overseeing risk-taking are unchanged in different periods (pre-crisis, crisis, or post-crisis periods). Our results suggest that conventional and Islamic banks should improve their corporate governance by enhancing the effectiveness of their audit committees and Sharia committees to constrain risk-taking. Any change in the structure of the audit committee or Sharia committee should take into account the economic cycle. In addition, regulators in countries that have two kinds of banks should have different guidelines for each kind of bank to restructure their corporate governance for purpose of better controlling risk-taking.

Since information about the audit committee and Sharia committee was not published by many banks, we did not analyze some characteristics of the audit committee and Sharia committee, such as the education or experience of their members. Moreover, our study only analyzed in an experimental way the role of the audit committee and the Sharia committee in overseeing risk-taking, even though they may also have a role in overseeing risk management activities. Future studies could analyze further characteristics of audit committee and the Sharia committee as a proxy for their effectiveness in affecting banks' risk-taking behavior or analyze the role of the audit committee and the Sharia committee on banks’ risk management activities.

## Declarations

### Author contribution statement

Quang Khai Nguyen: Conceived and designed the experiments; Performed the experiments; Analyzed and interpreted the data; Contributed reagents, materials, analysis tools or data; Wrote the paper.

### Funding statement

This research was supported by the University of Economics Ho Chi Minh City, Vietnam.

### Data availability statement

The authors do not have permission to share data.

### Declaration of interests statement

The authors declare no conflict of interest.

### Additional information

No additional information is available for this paper.
