# Supplementary material for: Oversight of bank risk-taking by audit committees and Sharia committees: conventional vs Islamic banks
Source: Heliyon. 2021 Aug 14;7(8):e07798. doi: 10.1016/j.heliyon.2021.e07798 (PMC8379450; doi:10.1016/j.heliyon.2021.e07798)
Supplement: Appendix A [file mmc1.docx]

**Appendix A: List of banks**

| Country | Conventional banks | Islamic banks |
| --- | --- | --- |
| Bahrain | Ahli United Bank | ABC Islamic Bank |
|  | Arab Bank plc | Al Baraka Banking |
|  | Bahrain Development Bank | Arab Islamic Bank |
|  | Housing Bank for Trade and Finance | Bahrain Islamic Bank |
|  | Bank of Bahrain and Kuwait | Bank Al-Khair |
|  | Credit Libanais | Citi Islamic Investment Bank |
|  | Future Bank | First Energy Bank |
|  | Habib Bank Limited | GFH Investment Bank |
|  | Mashreq Bank | Global Banking Corporation |
|  | National Bank of Bahrain | Ibdar Bank |
|  | United Bank Limited | Ithmaar Bank |
|  |  | Khaleeji Commercial Bank |
|  |  | Türkiye Finans Katılım Bankası A.Ş |
| Indonesia | Bank KB Bukopin | Bank Syariah Mandiri |
|  | Bank Bumi Arta | Muamalat Bank |
|  | Bank Capital Indonesia | Bank Indonesia |
|  | Bank Central Asia | Dubai Islamic Bank |
|  | Bank CTBC Indonesia |  |
|  | Bank Ganesha |  |
|  | Bank Index Selindo |  |
|  | Bank KEB Hana Indonesia |  |
|  | Bank Mayora |  |
|  | Bank Mestika Dharma |  |
|  | Bank MNC Internasional |  |
|  | Bank Multiarta Sentosa |  |
|  | Panin Bank |  |
|  | Bank Sinarmas |  |
|  | Bank Woori Saudara |  |
| Malaysia | Affin Bank Berhad | Affin Islamic Bank Berhad |
|  | Alliance Bank Malaysia Berhad | [Al Rajhi Bank](https://en.wikipedia.org/wiki/Al_Rajhi_Bank) |
|  | AmBank (M) Berhad | Alkhair International Islamic Bank Malaysia Berhad |
|  | [Bank of America Malaysia Berhad](https://www.bnm.gov.my/-/bank-of-america-malaysia-berhad) | Alliance Islamic Bank Berhad |
|  | Bank of China (Malaysia) Berhad | MBSB Bank Berhad |
|  | China Construction Bank (Malaysia) Berhad | AmBank Islamic Berhad |
|  | CIMB Bank Berhad | [Bank Islam Malaysia](https://en.wikipedia.org/wiki/Bank_Islam_Malaysia) |
|  | Citibank Berhad | [Bank Muamalat Malaysia](https://en.wikipedia.org/wiki/Bank_Muamalat_Malaysia) |
|  | Hong Leong Bank Berhad | Maybank Islamic Berhad |
|  | India International Bank (Malaysia) Berhad | OCBC Al-Amin Bank Berhad |
|  | Industrial and Commercial Bank of China (Malaysia) Berhad | Public Islamic Bank Berhad |
|  | Malayan Banking Berhad | RHB Islamic Bank Berhad |
|  | Mizuho Bank (Malaysia) Berhad |  |
|  | OCBC Bank (Malaysia) Berhad |  |
|  | Public Bank Berhad |  |
|  | [RHB Bank Berhad](https://www.bnm.gov.my/-/rhb-bank-berhad) |  |
|  | Standard Chartered Bank Malaysia Berhad |  |
|  | Sumitomo Mitsui Banking Corporation Malaysia Berhad |  |
|  | The Bank of Nova Scotia Berhad |  |
|  | United Overseas Bank (Malaysia) Bhd. |  |
| Pakistan | Askari Bank | Meezan Bank Limited |
|  | Allied Bank Limited | Soneri Mustaqeem Islamic Bank |
|  | Bank Alfalah | Dubai Islamic Bank |
|  | Bank Al Habib | Al Baraka Bank |
|  | Faysal Bank | Bank Alfalah Islamic |
|  | Habib Bank Limited | BankIslami Pakistan Limited |
|  | Habib Metropolitan Bank | National Bank of Pakistan |
|  | JS Bank | Bank of Punjab Islamic Banking |
|  | Samba Bank Limited |  |
|  | Silkbank Limited |  |
|  | Standard Chartered Pakistan |  |
|  | Soneri Bank |  |
|  | Summit Bank |  |
|  | United Bank Limited |  |
|  | MCB Bank Limited |  |
|  | Bank of Punjab |  |
|  | Sindh Bank |  |
|  | Industrial Development Bank |  |
| Singapore | Bank of East Asia | The Islamic Bank of Asia |
|  | CIMB Bank | Maybank |
|  | DBS Bank |  |
|  | Hongkong & Shanghai Banking Corporation |  |
|  | ICICI Bank |  |
|  | POSB Bank |  |
|  | RHB Bank |  |
|  | United Overseas Bank |  |
| Kuwait | Commercial Bank of Kuwait | Kuwait Finance House |
|  | Al Ali Bank | Warba Bank |
|  | Kuwait International Bank | Boubyan Bank |
|  | Industrial Bank of Kuwait | Al Ahli United Bank |
|  |  | Al-Rajhi Bank |
| Qatar | The Commercial Bank of Qatar | Qatar International Islamic Bank |
|  | Qatar Industrial Development Bank | - Qatar Islamic Bank. |
|  | Arab Bank | - Barwa Bank. |
|  | Mashreq Bank Qatar |  |
|  | Standard Chartered Bank Qatar |  |
| Saudi Arabia | National Commercial Bank | Al Jazeera Bank |
|  | Saudi British Bank | Al-Bilad Bank |
|  | Saudi Investment Bank |  |
|  | Banque Saudi Fransi |  |
|  | Riyad Bank |  |
|  | Alawwal bank |  |
|  | Samba Financial Group |  |
| UAE | Dubai Finance Bank | Dubai Islamic Bank |
|  | Bank of Baroda | Abu Dhabi Islamic Bank |
|  | Commercial Bank of Dubai | Emirates Islamic Bank |
|  | National Bank of Fujairah | Sharja Islamic bank |
|  | National Bank of Umm Al-Qaiwain |  |
| Bangladesh | AB Bank Limited | Islami Bank Bangladesh Ltd |
|  | Bangladesh Commerce Bank Limited | Shahjalal Islami Bank |
|  | Bank Asia Limited | ICB Islamic Bank |
|  | BRAC Bank Limited | Social Islami Bank Limited |
|  | Citizens Bank PLC |  |
|  | Jamuna Bank Limited |  |
|  | Meghna Bank Limited |  |
|  | Mercantile Bank Limited |  |
|  | Midland Bank Limited |  |
